# Neuronal Activation in the Periaqueductal Gray Matter Upon Electrical Stimulation of the Bladder

**DOI:** 10.3389/fncel.2018.00133

**Published:** 2018-05-18

**Authors:** Céline Meriaux, Ramona Hohnen, Sandra Schipper, Aryo Zare, Ali Jahanshahi, Lori A. Birder, Yasin Temel, Gommert A. van Koeveringe

**Affiliations:** ^1^School for Mental Health and Neuroscience (MHeNS), Maastricht University, Maastricht, Netherlands; ^2^European Graduate School of Neuroscience (EURON), Maastricht, Netherlands; ^3^Department of Urology, Maastricht University Medical Center, Maastricht, Netherlands; ^4^Department of Neurosurgery, Maastricht University Medical Center, Maastricht, Netherlands; ^5^Department of Medicine, University of Pittsburgh School of Medicine, Pittsburgh, PA, United States

**Keywords:** periaqueductal gray, bladder, sensory, brain-bladder, lower urinary tract symptoms

## Abstract

Reflexes, that involve the spinobulbospinal pathway control both storage and voiding of urine. The periaqueductal gray matter (PAG), a pontine structure is part of the micturition pathway. Alteration in this pathway could lead to micturition disorders and urinary incontinence, such as the overactive bladder symptom complex (OABS). Although different therapeutic options exist for the management of OABS, these are either not effective in all patients. Part of the pathology of OABS is faulty sensory signaling about the filling status of the urinary bladder, which results in aberrant efferent signaling leading to overt detrusor contractions and the sensation of urgency and frequent voiding. In order to identify novel targets for therapy (i.e., structures in the central nervous system) and explore novel treatment modalities such as neuromodulation, we aimed at investigating which areas in the central nervous system are functionally activated upon sensory afferent stimulation of the bladder. Hence, we designed a robust protocol with multiple readout parameters including immunohistological and behavioral parameters during electrical stimulation of the rat urinary bladder. Bladder stimulation induced by electrical stimulation, below the voiding threshold, influences neural activity in: (1) the caudal ventrolateral PAG, close to the aqueduct; (2) the pontine micturition center and locus coeruleus; and (3) the superficial layers of the dorsal horn, sacral parasympathetic nucleus and central canal region of the spinal cord. In stimulated animals, a higher voiding frequency was observed but was not accompanied by increase in anxiety level and locomotor deficits. Taken together, this work establishes a critical role for the vlPAG in the processing of sensory information from the urinary bladder and urges future studies to investigate the potential of neuromodulatory approaches for urological diseases.

## Introduction

Overactive bladder symptom complex (OABS) is one of the most frequently encountered urological disorders. In the western world, the number of people suffering from OABS has been evaluated at almost 100 million (Stewart et al., 2003; Irwin et al., 2006; Hashim and Abrams, 2007). The complex is defined as a medical condition encompassing urgency, increased daytime frequency, nocturia and urgency urinary incontinence (i.e., involuntary loss of urine associated with urgency) either isolated or in any combination (Abrams et al., 2003). Although this is not considered as a severe health condition, it has a significant impact on personal autonomy, self-esteem, quality of life, quality of sleep and mental health and it creates an enormous economic burden (Irwin et al., 2006; Onukwugha et al., 2009).

Nowadays, the first line treatment for OABS relies on the application of antimuscarinic drugs (Madhuvrata et al., 2012; Veenboer and Bosch, 2014). The second line therapies include botulinum toxin injection into the detrusor muscle (Chapple et al., 2013; Nitti et al., 2013; Mangera et al., 2014; Giannantoni et al., 2015), sacral neuromodulation (Peters et al., 2009, 2010; Sukhu et al., 2016) or invasive surgical interventions such as an augmentation cystoplasty (Reyblat and Ginsberg, 2008). However, these therapeutic interventions are only effective in a subset of patients and their effectiveness is limited in the remainder. One of the key elements of OABS is related to faulty sensory signaling of the filling status of the bladder. Knowledge about structures within the central nervous system involved in the micturition pathway can aid in developing novel rationally-designed treatment modalities and can help to identify patients, which are good candidates for specific lines of treatment.

The two functions of the lower urinary tract (LUT), i.e., urinary bladder and urethra, to store and periodically expel urine, are dependent upon central and local neural circuits located in the brain, the spinal cord and the peripheral ganglia (Fowler, 2006; Fowler et al., 2008; Birder et al., 2010; de Groat et al., 2015). Altered reflexes and sensory perceptions within the bladder—brain axis have emerged as a generally accepted model to explain pathologies such as OABS (Abrams, 2003). The modulation of sensory afferent functioning by means of neuromodulation could be considered a promising option for the treatment of OABS. However, in order to reach maximal efficacy and minimal side effects targets need to be identified.

One vital structure in the central pathway of the micturition pathway is the periaqueductal gray matter (PAG). The PAG receives ascending sensory fibers from the bladder, which pass through the dorsal horn of the spinal cord. The PAG is connected to the pontine micturition center (PMC; also called Barrington’s nucleus). The PAG, in turn, is connected to other supraspinal structures of which some belong to the limbic system (such as the amygdala, insula) but also the prefrontal cortex and the thalamus (Vizzard et al., 1995; Blok et al., 1997, 1998; Athwal et al., 2001; Griffiths et al., 2005; Griffiths and Tadic, 2008; Fowler and Griffiths, 2009; Tai et al., 2009).

It is generally believed that all ascending signals (such as mechanosensation, Griffiths and Tadic, 2008) are relayed through the PAG to the PMC. The PMC is the site for the initiation of the micturition reflex (Blok and Holstege, 1994; Matsuura et al., 2000; Taniguchi et al., 2002). Therefore the PAG is considered to be at the crossroads of ascending sensory information and inputs from higher centers that modulate these processes and plays the role of coordination center (Blok et al., 1995). Inputs from higher cortical systems are related to emotions, i.e., anxiety, and interpretation of social aspects (Mantyh, 1982; Griffiths et al., 2007; Griffiths and Fowler, 2013).

Based on neuroanatomical and cytoarchitectural features the PAG has been subdivided into four longitudinal columns spanning in the rostro-caudal direction, which are classified as the dorsomedial, dorsolateral, lateral and ventrolateral column (Beitz, 1985; Carrive, 1993; Bandler and Shipley, 1994; Linnman et al., 2012; Menant et al., 2016). Besides, their neuroanatomical distinction, these columns are also involved in different physiological processes. It has been described that the PAG is involved in autonomic (respiration and cardiovascular functions) as well as pain related functioning.

Several studies have investigated neuronal connections to and from the PAG by means of imaging or neuronal tracing. However, functional connectivity in response to afferent sensory stimuli and in particular stretch-related mechanoperception have—to our knowledge—never been investigated in a physiological manner.

Therefore, the present manuscript describes three groups of experiments. First, we identified an optimal stimulus applied to the bladder, in order to provoke sensory afferent stimulation in the absence of voiding. Second, we evaluated what behavioral consequences this stimulus has. We evaluated these consequences in the light of the different functions (i.e., anxiety and pain) that the PAG is mediating. Third, we investigated the pattern of neuronal activation in different parts of the central nervous system and in particular in the PAG. We did so by localizing and quantifying the amount of cFos positive cells in the various columns of the PAG, the PMC, the locus coeruleus, and several nuclei of the sacral spinal cord after urinary bladder stimulation in freely moving rats, with the previously optimized parameters.

## Materials and Methods

### Subjects

Adult, male *Sprague-Dawley* rats (body weight 300–350 g at the time of surgery) from Charles River were housed in the Central Animal Facility (Maastricht University, Netherlands). Rats were individually placed in Makrolon™ cages. Housing conditions for temperature (21 ± 1°C) and light (reversed 12 h light/dark cycle, 7 a.m.–7 p.m. lights off, music on) were controlled and food and water were provided *ad libitum*. All experimental animal procedures were executed during the dark phase, under red light, when rodents are most active.

### Bipolar Stimulation Electrode Implantation

For the purpose of these experiments we developed a novel methodology for the electrical stimulation of the detrusor muscle. The idea is derived from the principle of transurethral intravesical electrostimulation (IVES), which has been used to initiate non-voiding contractions, which result in the depolarization of afferent sensory nerve fibers and subsequently a strong centrally-induced detrusor contraction (Madersbacher, 1990; Ebner et al., 1992; Buyle et al., 1998; Jiang, 1998; Streng et al., 2006).

We implanted rats chronically with a custom-made bipolar stimulation construct, which consists of two pacing wires (Streamline™, Medtronic, France) that were surgically implanted in the bladder wall. The wires were tunneled transdermally and a platform attached to the head was used as plug in order to easily connect an external power source.

For the surgical procedure, anesthesia was achieved by isoflurane inhalation (IsoFlo^®^, Abbott Laboratories Ltd., Great Britain; induction: 4%, maintenance: 1.5%) and the temperature was monitored using a rectal temperature probe and maintained within one degree of 37°C using a heating blanket. The abdomen, the neck and the head were shaved and disinfected using Betadine^®^. A small incision was made in the neck of the animal to subcutaneously tunnel the wires. A midline laparotomy was made to expose the urinary bladder and insert the exposed pacing wires between the serosa and the muscular layer. Two configurations have been used for the implantation of the bipolar electrode: (i) one pacing wire at the level of the dome and the other one at the level of the neck of the bladder, close to the urethra (hereafter abbreviated as: D-N); and (ii) both pacing wires at the level of the neck of the bladder (hereafter abbreviated as: N-N). To keep the electrode in place, to insulate the tips and protect the surrounding tissues from scratching, pieces of polyethylene tubing were sealed at the free end of wires. The abdominal incision was then closed. After installing the rat in the stereotactic apparatus (Stoelting model 51950, Stoelting Co., Wood Dale, IL, USA), a dermal incision and periostomy were performed to facilitate anchoring of the construct to the skull by using miniature screws (stainless steel, 1 × 2 mm). The platform was positioned on top of them and fixed with dental cement (Paladur, Heraeus Kulzer GmbH, Germany). For cFos immunostaining and behavioral experiments, a recovery period of 2 weeks was given before stimulation and testing.

### Optimization of Stimulation Parameters

#### Electrical Stimulation of the Urinary Bladder and *in Vivo* Cystometry

To evaluate which parameters were optimal for electrical stimulation of the urinary bladder, an experiment was performed combining electrical stimulation and *in vivo* cystometry in five rats under terminal anesthesia with urethane (1.5 g/kg). Besides the two electrodes, a catheter was implanted at the dome of the bladder in order to measure intravesical pressure. For this reason, electrodes were implanted at opposing sides in the lateral bladder wall for all animals in this experiment. The catheter was made of PE-50 tubing (BD Medical, USA) with a cuff and inserted into the bladder dome during the laparatomy through the low abdominal incision and held in place with a purse-string suture.

After the recovery period, the bladder catheter was connected via a T-tube to a pressure transducer and an infusion pump. Saline solution was infused into the bladder at a flow rate of 90 μl/min. The bladder was filled with saline up to 0.6 ± 0.2 ml and the grade of bladder filling was kept stable during the stimulation protocol. The intravesical pressure was recorded continuously using a disposable IBP pressure transducer (DPT-6000, Codan pvb Medical GmbH, Germany) and the MP150 data acquisition system equipped with an amplifier module and acquired with the AcqKnowledge software (AcqKnowledge 4.2 version, BIOPAC system Inc., Goleta, CA, USA). Simultaneously, stimulation was applied by connecting animals to a stimulus isolator (DS100, WPI Europe, Germany) driven by a digital stimulator (DS8000, WPI Europe, Germany). After bladder stabilization, electrical stimulation was given with constant current biphasic square wave pulses of 0.5 ms. Studies showed that sensory nerves were more effectively stimulated with a pulse width of 0.5 ms whereas motor fibers were preferentially activated at shorter pulse duration (0.05–0.4 ms; Jiang and Lindström, 1996; Tsui, 2008). Additionally, longer pulse duration (>2 ms) might cause tissue damage.

We aimed at establishing optimal stimulation frequency (ranging from 10 Hz to 200 Hz) and intensity (ranging from 2 mA to 3 mA) in order to induce intravesical pressure augmentation. In order to prevent confounding, a randomized stimulation paradigm with different frequencies was applied with electrical stimulations lasting 30 s followed by a stimulation-off period of 60 s to allow intravesical pressure to return to baseline.

After the experiment rats were sacrificed by means of decapitation and no further post-mortem analysis took place.

### Stimulation Induced Behavioral Changes

To assess locomotor activity, anxiety and voiding pattern changes in rats during electrical stimulation of the urinary bladder, the following behavioral tests were performed: Open Field (OF), Elevated Zero Maze (EZM) and Voiding behavior tasks (VBTs). Five rats underwent all behavioral tests in a repeated design after a period of habituation to the handler and experimental environment at the following time points: at baseline prior to the surgical procedure (Control condition), 2 weeks after the electrode implantation in the presence (Stimulated condition) and absence (Sham condition) of electrical stimulation. For all behavioral experiments the electrodes were implanted in the D-N configuration.

At the end of the experiments rats were sacrificed and their tissue was not used for further *post-mortem* investigations due to the potential confounding influence of repeated stimulation.

#### Open Field Task

Spontaneous locomotor activity was measured in the OF, which consists of a large Plexiglas square arena (100 × 100 cm) with 40 cm high transparent Plexiglas walls and a dark Plexiglas floor. The trials were recorded under low light conditions and the total distance moved was automatically assessed via a video camera connected to a video tracking system (Ethovision, Noldus, Netherlands).

#### Elevated Zero Maze Task

Anxiety was evaluated in the EZM, a black plastic circular runway (100 cm in diameter, 10 cm path width) placed 70 cm above the floor level and equally divided into two opposite open and two opposite enclosed parts with 50 cm high side walls. To prevent falls, a 7-mm high rim surrounds the open arms. Animals were placed into one of the open arms facing a closed part and allowed to explore the maze for 5 min. Time spent in the open parts was automatically quantified by means of the Ethovision setup under low light conditions through an infrared video camera connected to a video tracking system.

#### Voiding Behavior

Voiding behavior was assessed with a filter paper assay in combination with video-assisted tracing as previously described for mice (Biallosterski et al., 2015). We adapted the procedure to the body size of rats by changing the size of the cage and re-calculating a standard curve for estimating the relation between spot size and volume of urine voided.

All rats were assessed at the same time of the day to minimize the effect of the circadian cycle on micturition frequency and volume. Testing started 24 h after a habituation period lasting for 5 h in which the animal was placed into the cage in which the measurement took place.

Animals were placed on an elevated mesh floor, in a standard cage with its floor covered by filter paper. For 1-h, voiding behavior of freely moving rats was observed using a video camera placed below the cage. Thereafter, the number of spots, and the volume of voided urine were assessed as indicators for the voiding frequency, and the total of discharged urine. For this purpose, the filter paper was exposed to ultraviolet light and spot quantity and size were measured in ImageJ.

### Neuronal Activation in the Central Nervous System

#### Experimental Groups

Rats were randomly assigned to one of the five experimental groups: (I) Control group without electrode implantation (*n* = 6), (II) Sham D-N group (*n* = 6), (III) Sham N-N group (*n* = 6), (IV) Stim D-N group (*n* = 7) and (V) Stim N-N group (*n* = 7). Sham animals underwent the same surgical procedure with electrode implantation and were connected to the stimulator without stimulation being performed.

#### cFos Immunohistochemistry

Following a 2-week recovery period, 1-h continuous electrical stimulation of the bladder in freely moving rats was administrated using the previously optimized stimulation parameters.

For this purpose, rats were placed in custom-made cages that had equal dimensions as their home cages. These cages were modified in a way that the top of the cage allowed the connection of the head-mounted connector to the stimulator. Before the start of the stimulation rats were habituated for 1 h.

Ninety minutes after the end of stimulation (Rodella et al., 1998; Baulmann et al., 2000; Haller et al., 2006; Arout et al., 2014), rats were transcardiac perfused with Tyrode’s buffer and with fixation solution containing 4% paraformaldehyde, 15% picric acid, 0.05% glutaraldehyde in 0.1 M phosphate buffer (pH 7.6; Somogyi and Takagi, 1982). Thirty-two brains and six spinal cords (Control *n* = 2, Sham D-N *n* = 2 and Stim D-N *n* = 2) were removed rapidly and post-fixed in fresh fixative solution (Fixation solution without glutaraldehyde) for 2 h at 4°C prior to overnight immersions in an ascending series of sucrose (10% and 20% sucrose in 0.1 M phosphate buffer) at 4°C. Finally, tissue was snap-frozen in solid carbon dioxide and stored at −80°C until being sectioned coronally into 30 μm serial slices using a cryostat (Leica, Germany). Brains sections were collected into cups to be processed immunohistochemically as free-floating sections whereas spinal cord sections were mounted on gelatin-coated glass slides. Serial sections were kept at −80°C before staining.

We applied two different ways of visualizing cFos. For the brain sections, we used the indirect immunohistochemical (3,3′-Diaminobenzidine, DAB) staining method employing amplification by means of streptavidin and nickel-diaminobenzidine chromogen enhancement according to previous protocols (Tang et al., 2018) whereas for spinal cord sections we chose fluorescent visualization. Because brain sections are known for possessing auto-fluorescence after formaldehyde fixation (Clancy and Cauller, 1998; Schnell et al., 1999; Spitzer et al., 2011) and we expected low cFos epitope expression, we opted for the indirect immunohistochemical visualization in the brain. In the spinal cord, the secondary aim was to perform double labeling techniques, which is facilitated by the use of fluorophores.

Brain sections containing the PAG (Bregma AP: −6.3 to −8.3 mm) and the pons (Bregma: AP: −9.6 to 10.0 mm) were incubated overnight at 4°C with monoclonal mouse anti-cFos primary antibody (1:2000 dilution, Santa Cruz Biotechnology Inc., Santa Cruz, CA, USA), washed with Tris-buffered saline (TBS) and TBS-Triton X-100, and then incubated with the biotinylated donkey anti-mouse secondary antibody (1:400 dilution, Jackson Immunoresearch Laboratories, USA) for 1 h. This was followed by exposure to avidin-biotin-peroxidase complex (1:800, Elite ABC-kit, Vestastatin, Vector Laboratories, USA) for 2 h. The staining was visualized using a DAB solution and nickel intensification. Brain sections were mounted on gelatin-coated glass slides, dehydrated and cover-slipped with Pertex mounting medium (Histolab Products AB, Sweden). Spinal cord sections (L6 to S2 segments) were incubated overnight at room temperature with monoclonal mouse anti-cFos primary antibody (1:2000 dilution, Santa Cruz Biotechnology Inc., USA) and rinsed following the same protocol as for brain sections. Subsequently, a 2-h incubation with the secondary antibody Alexa Fluor^®^ 488-conjugated donkey anti-mouse (1:100 dilution, Thermo Fisher Scientific, NY, USA) was followed by three washing steps with TBS and by coverslipping in 80% glycerol/TBS).

Photographs of the stained brain sections were taken (4× and 10× magnification) using a U-CMAD-2 digital camera mounted on an Olympus AX70 bright-field microscope (analySIS, Imaging System, Germany) and evaluated with ImageJ analysis software (ImageJ software, NIH, USA). We assessed results for the caudal and rostral regions of the PAG separately. In the rostral part of the PAG no ventrolateral (vl) column can be found and therefore results are based on the dorsomedial (dm), dorsolateral (dl) and lateral column of the PAG. In the caudal portion of the PAG all four columns are present (Lawrenson et al., 2017; Supplementary Figure S1). Columns were delineated from similar Bregma levels of two rostral (AP: −6.7 mm and −7.0 mm) and two caudal (AP: −7.7 mm and −8.0 mm) PAG images per rat. The locus coeruleus (LC) and PMC were delineated on the two hemispheres from one pontine region image per rat.

We assessed the quantity of cFos positive cells in the brain in the following way. First, we acquired pictures from the region of interest with a low magnification (4×). Second, we delineated the region or columnar areas with a higher magnification (10×), which resulted in an area, which was used for the quantification of the cell number (Supplementary Figure S2). In all cases, the delineation was based on standard anatomical hallmarks for the PAG (i.e., an angle of the circumference) or corresponding histological stainings of the consecutive section (Nissl staining for the PMC and LC) and the anatomical descriptions of these regions according to the standard rat brain atlas of Paxinos and Watson (2007). Consequently, a thresholding method was applied in the delineated sections, which identified all cells, which had an intensity that exceeded the background (Hescham et al., 2015). Cells were then manually counted by two independent observers. Observers were blinded for treatment groups. The values were expressed as cells per area to correct for potential differences in the delineation of the areas and expressed as cells/mm^2^.

A different workstation was used for the acquisition of pictures for the activation of cFos positive cells in the segments L6-S2 of the spinal cord. The analysis was performed online using a stereology workstation, i.e., a modified Olympus BX50 fluorescence microscope controlled by the Stereo Investigator software (MBF Bioscience, Williston, VT, USA). Delineations of the regions of interest (Laminae I to III of the dorsal horn, Medial gray region with the DCM, Lateral gray subdivision including the SPN and the ventral horn) were made on the microscopic images displayed on a monitor using 4× objective and adjusted at a higher magnification (10×). Within the delineated areas, the total number of cFos positive cells was evaluated using a 40× objective and the optical fractionator of the Stereo Investigator software by manually counting cFos positive cells. Because of the limited number of sections per region of interest, we did not extrapolate the data into a 2D volume but chose to present results per slice.

### Electrode Localization

The location of the electrode was assessed in a sample of the implanted animals by performing a macroscopic *post-mortem* examination. This was accompanied by the microscopic assessment of cryosection of the bladder. For this purpose, bladder strips were prepared, which were cut and rapidly assessed under bright field illumination. No staining procedures or quantifications were performed. This method revealed no overt signs of fibrosis, dis-location or inflammation in the assessed bladders.

### Statistics

Data were presented as mean ± SEM values. For the evaluation of stimulation parameters, a one-way ANOVA was employed, followed by a Fisher PLSD *post hoc* test. Histological and behavioral data were analyzed statistically with inter-group comparisons using *t*-test. All statistical analyses were performed with Sigma Plot 11.0 version for Windows (Systat Software). *P-values* lower than 0.05 were considered statistically significant.

### Ethical Approval

All procedures were in line with local and national guidelines and laws on animal welfare. This study was carried out in accordance with the recommendations of Guidelines for Ethical Conduct in the Care and Use of Nonhuman Animals in Research. The protocol was approved by the “Animal ethical committee of Maastricht University” and all procedures performed were in accordance with ethical standards of Maastricht University.

## Results

### Cystometric Investigation for Electrical Stimulation Parameter Optimization

**Figure 1:**
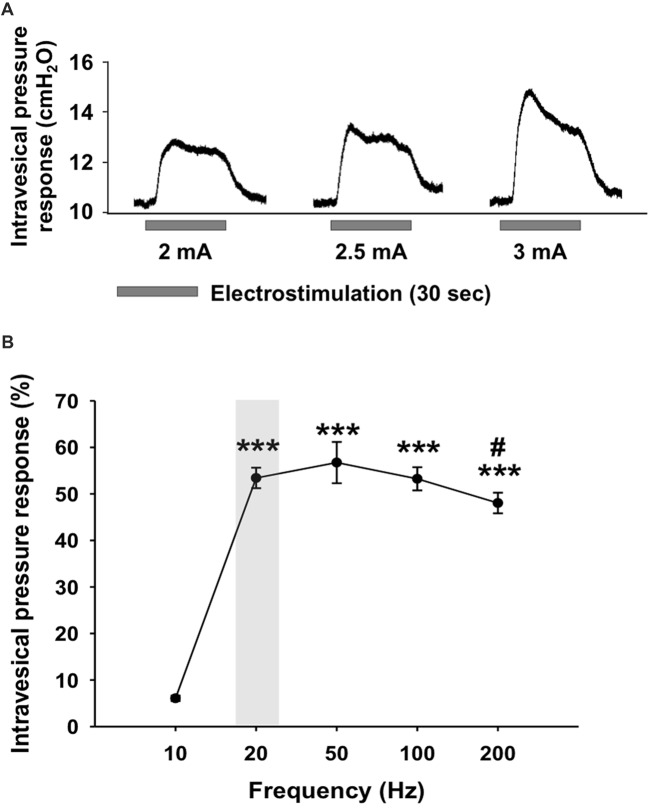
Intravesical pressure responses during electrical stimulation of the urinary bladder. **(A)** Representative intravesical pressure responses during electrical stimulation with a frequency adjusted to 20 Hz and an intensity set at 2, 2.5 and 3 mA. **(B)** Intravesical pressure responses during electrical stimulation with intensity of 2 mA and variable frequency from 10 Hz and 200 Hz. The intravesical pressure responses were significantly lower for 10 Hz frequency compared to frequencies higher than 20 Hz. The increase in intravesical pressure following a stimulation with 200 Hz was lower than with 50 Hz. Values are represented as means ± SEM *n* = 5. One-way ANOVA, *F*_(4,24)_ = 61.175, *** vs. 10 Hz, *p* < 0.001; ^#^ vs. 50 Hz, *p* = 0.034.

The effect of different combinations of parameters (i.e., frequency and intensity) on the induced non-voiding detrusor contraction was evaluated to find the most effective setting for 1-h continuous electrical stimulation of the bladder with the bipolar electrode. Pressure responses were observed during electrical stimulation when the current intensity was higher than 2 mA, for a frequency range of 20–200 Hz. For 10 Hz-stimulations, an intensity of 3 mA was required to trigger an intravesical pressure increase. In all animals, the pressure increase, and the stimulus amplitude were positively correlated (Figure 1A). In five animals, the maximal pressure response (4.55 cm H_2_O ± 1.07) was obtained with a current intensity of 3 mA. The response to electrical stimulation of the bladder with a current intensity of 2 mA was characterized by a rise in pressure to reach a plateau and then a decrease towards the baseline (Figure 1A). Higher current intensities (2.5 mA and 3 mA) elicited an acute increase of pressure, showed as a sharp peak, and followed by a decrease of pressure to the plateau level (Figure 1A). However, in order to avoid tissue injury and at the same time to avoid inducing a micturition contraction during long-term electrical stimulation, the current intensity of 2 mA, the lowest current intensity capable of inducing a response, was chosen. Because the intravesical pressure responses could depend on the position of the electrode, we standardize the statistical analysis, by normalizing the data and expressed these as percentage of the maximal intravesical pressure response per animal. With the current intensity set at 2 mA, the intravesical pressure responses obtained upon electrical stimulation were frequency-dependent (One-way ANOVA, *F*_(4,24)_ = 61.175, *p* < 0.001; Figure 1B). With a frequency of 10 Hz, low intravesical pressure responses were induced (6.08% ± 1.28). At 20 Hz, the response was maximal (54.4% ± 5.2) and was not significantly different from the responses found using higher frequencies (NS, 20 vs. 50 Hz, *p* = 0.396; 20 vs. 100 Hz, *p* = 0.963; 20 vs. 200 Hz, *p* = 0.175, Fischer PLSD). The observed response to electrical stimulation with 200 Hz was lower than with the intermediate frequencies (50 Hz vs. 200 Hz, 56.7% ± 4.4 vs. 48.04% ± 2.2, *p* = 0.034). To minimize tissue damage due to continuous electrical stimulation, we opted for the frequency of 20 Hz, the lowest frequency stimulation giving a maximal pressure response. Therefore, the optimal combination of both parameters inducing an adequate sensation inducing contraction of the bladder following electrical stimulation was determined as having a frequency of 20 Hz and an amplitude of 2 mA and has been used for the subsequent set of experiments.

### Behavioral Measurements During Electrical Stimulation of the Urinary Bladder

After the electrode implantation, in both stimulated and non-stimulated animals, characteristic behavioral signs of visceral pain were assessed. No behavioral modifications including lacrimation, piloerection, tail hyperextension, abdominal contraction, arched posture, lower abdomen licking and backward withdrawal movements were displayed in neither the sham nor the stimulated conditions.

Previous works suggested that stress and pain are related to the activation of specific columns in the PAG (Carrive, 1993; Bandler and Shipley, 1994). For instance, the dorsal PAG is linked to avoidance behavior, the lateral PAG is correlated to fight and flight whereas the ventrolateral PAG is associated with freezing and immobility. To confirm that the neuronal activation observed in the PAG related to bladder-related functioning and was not triggered by confounding behaviors potentially generated by electrode implantation or electrical stimulation of the urinary bladder, the locomotor behavior was evaluated in the OF. Analysis of the behavioral data in the OF showed that the total distance traveled was not significantly different in stimulated animals compared to sham animals (Sham vs. Stim, 5063.8 cm ± 135.9 vs. 5271.3 cm ± 559.2, *p* = 0.753). Therefore, neither the implantation of the bipolar electrode nor the electrical stimulation of the bladder alters spontaneous locomotor activity.

Moreover, animals were tested in the EZM to determine whether anxiety level was affected by the experimental procedure. No significant effect was found between the control and sham conditions (*t*-test, NS, *p* > 0.05). Moreover, the time spent in open arms of the EZM was comparable between the sham and stimulated conditions (Sham vs. Stim, 95.7 ± 34.8 vs. 106.1 ± 18.6, *p* = 0.805) indicating no change in terms of anxiety-like behavior.

**Figure 2:**
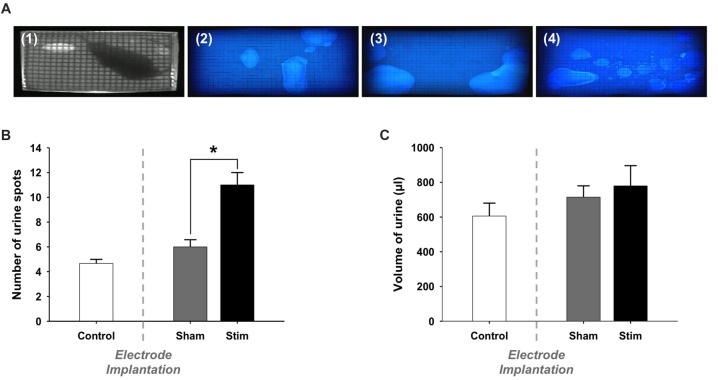
Voiding behavior: Voiding parameters. **(A)** Representative photographs of (1) an extract from the video recorded during a voiding behavior task (VBT) and filter papers from the same rat (2) before the surgery (Control), (3) after the electrode implantation (Sham) and (4) during electrical stimulation of the bladder (Stim). **(B)** Number of urine spots. During the stimulation, rats voided more frequently than when the stimulation was switched-off. Values are represented as means ± SEM. Control *n* = 5, Sham *n* = 5 and Stim *n* = 5. *t*-test, Sham vs. Stim, *p < 0.05. **(C)** Total volume of voided urine. The total volume of urine spots was not significantly larger in stimulated condition compared to sham and control conditions.

Also, we assessed whether stimulation changed voiding behavior (Figure 2A). The quantification of voiding spots reveals no following electrode implantation (Control vs. Sham, 4.7 ± 0.3 vs. 6.0 ± 0.6, *p* = 0.116; Figure 2B). However, electrical stimulation of the bladder caused a significant increase of voiding frequency compared to the un-stimulated condition (Sham vs. Stim, 6.0 ± 0.6 vs. 11.0 ± 1, *p* = 0.012).

We estimated the total volume of voided urine during the VB by means of a volumetric calibration, which revealed a linear relation between spot size and volume voided. Video recordings were used to discriminate between overlapping urine spots. We found that the total volume of voided urine was comparable before and after surgery (Control vs. Sham, 0.61 ml ± 0.08 vs. 0.72 ml ± 0.07, *p* = 0.33; Figure 2C). Moreover, the total volume of voided urine was not altered in stimulated rats (Sham vs. Stim, 0.72 ml ± 0.07 vs. 0.78 ml ± 0.12, *p* = 0.652). The duration of the test we used resulted in a few voids at baseline. Therefore, we could not reliably quantify whether changes in the voiding pattern occurred in the different experimental conditions.

In summary our behavioral results indicate that there were no factors such as anxiety or pain, that could explain the activation of PAG columns. However, stimulation induced a higher frequency of voiding with smaller volumes per void.

### Effect of Electrical Stimulation of the Urinary Bladder on cFos Immunoreactivity in the PAG

**Figure 3:**
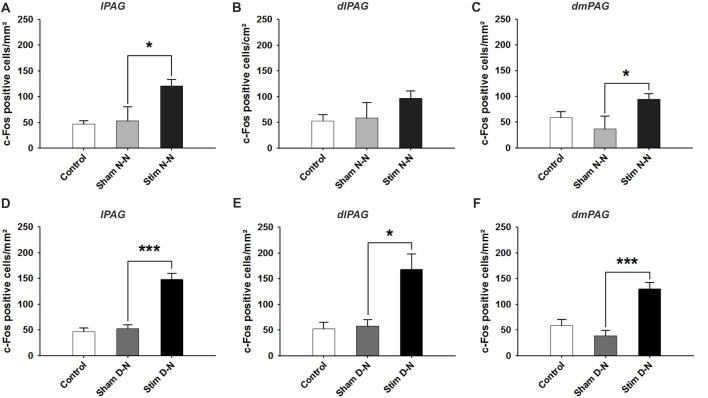
Expression of cFos immunoreactive cells in the rostral periaqueductal gray matter (PAG). Cumulative data showing the means and SEM. of cFos expression for N-N group in the rostral **(A)** lPAG, **(B)** dlPAG, **(C)** dmPAG and for D-N group in the rostral **(D)** lPAG, **(E)** dlPAG, **(F)** dmPAG. Control *n* = 12, Sham N-N *n* = 12, Sham D-N *n* = 12, Stim N-N *n* = 14 and Stim D-N *n* = 14. *t*-test, *p < 0.05 and ***p < 0.001.

**Figure 4:**
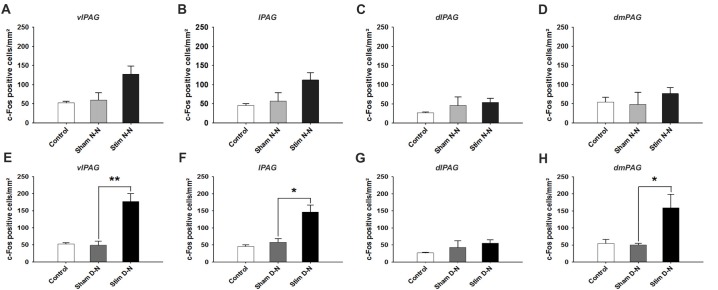
Expression of cFos immunoreactive cells in the caudal PAG. Cumulative data showing the means and SEM of cFos expression for N-N animals in the caudal **(A)** vlPAG, **(B)** lPAG, **(C)** dlPAG, **(D)** dmPAG and for D-N animals in the caudal **(E)** vlPAG, **(F)** lPAG, **(G)** dlPAG, **(H)** dmPAG. Control *n* = 12, Sham N-N *n* = 12, Sham D-N *n* = 12, Stim N-N *n* = 14 and Stim D-N *n* = 14. *t*-test, *p < 0.05 and **p < 0.005.

The cFos expression and its topographical localization in the PAG following electrical stimulation of the bladder were investigated to detect induced neuronal activation. Sham groups were used to test whether the presence of the electrode or the procedure of the electrode implantation *per se* were able to induce cFos expression in the PAG. In all sham groups, the cFos expression did not differ from control animals (Control vs. Sham, *t*-test, NS, *p* > 0.05; Figures 3, 4). No effect of electrode implantation (control vs. sham), regardless of pacing wires configuration, i.e., N-N vs. D-N, was observed on neuronal activation in any column of the PAG. Electrical stimulation of the urinary bladder upregulated cFos expression in several PAG columns compared to sham animals. This effect will be discussed separately for the rostral and caudal part of the PAG below.

### Activation of Neurons in the Rostral Part of the PAG

In both electrode configurations there was a significant increase of cFos positive cells in all columns of rostral part of the PAG except for a non-significant difference between sham and stimulated animals in the N-N configuration in the dl PAG. For N-N stimulated animals, the number of cFos positive cells in the lPAG and dmPAG was higher compared to sham control animals (Sham vs. Stim, lPAG and dmPAG, *p = 0.044 and *p = 0.046, respectively; Figures 3A–C). Electrical stimulation of the urinary bladder in D-N group increased the number of activated neurons in the lPAG, dlPAG and dmPAG (Sham vs. Stim, lPAG, dmPAG and dmPAG, ***p < 0.001, *p = 0.044 and ***p = 0.001, respectively; Figures 3D–F).

### Activation of Neurons in the Caudal Part of the PAG

**Figure 5:**
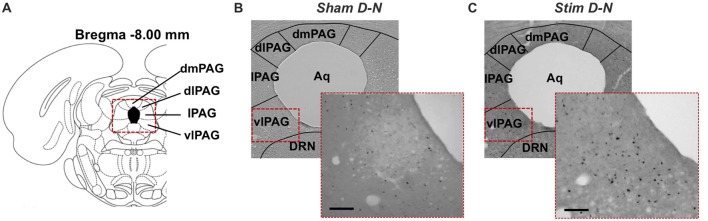
Distribution of cFos immunoreactive cells in the caudal PAG. **(A)** Coronal diagram of the rat brain at corresponding Bregma level AP −8.00 mm (Rat brain atlas, Paxinos and Watson, 2007). Representative low power photomicrographs of coronal brain sections stained for cFos showing the PAG of **(B)** non-stimulated (Sham) rat and **(C)** stimulated rat (Stim) with bipolar stimulation electrode implanted in the bladder wall at the dome and neck levels. The higher power photomicrographs in the lower right corners focus on the central region of the vlPAG column. Aq, aqueduct; DRN, dorsal raphe nucleus; dlPAG, dorsolateral PAG; dmPAG, dorsomedial PAG; lPAG, lateral PAG; vlPAG, ventrolateral PAG. Scale bar = 100 μm.

For N-N electrode configuration, no statistically significant difference in the number of cFos positive neurons was found in the caudal PAG between stimulated and sham groups (*t*-test, NS, *p* > 0.05; Figures 4A–D). For D-N electrode implantation, cFos counts in the ventrolateral subdivision of the caudal PAG were significantly greater in stimulated compared to sham rats (Sham vs. Stim, 48.8 ± 11.9 vs. 177.4 ± 23.1 cells/mm^2^; *p* = 0.003; Figure 4E). This was also the case for lateral and dorsomedial subdivisions of the caudal PAG (Sham vs. Stim, lPAG and dmPAG, *p = 0.012 and *p = 0.024, respectively; Figures 4F,H). The strongest effect size was seen in the caudal vlPAG compared to the other columns of the PAG (Figure 4). Moreover, the ventrolateral aspects of the caudal PAG close to the aqueduct in stimulated compared to sham D-N rats seemed to be more densely populated with activated neurons (Figure 5). Indeed, this observation was quantitatively confirmed by cFos cells counting, showing that the majority (53.8% ± 7.8%) of activated neurons were located in the central one third closest to the aqueduct of the vlPAG area. We did not observe the clustering of neurons in other columns.

### Effect of Electrical Stimulation of the Urinary Bladder on cFos Immunoreactivity in the Pons

**Figure 6:**
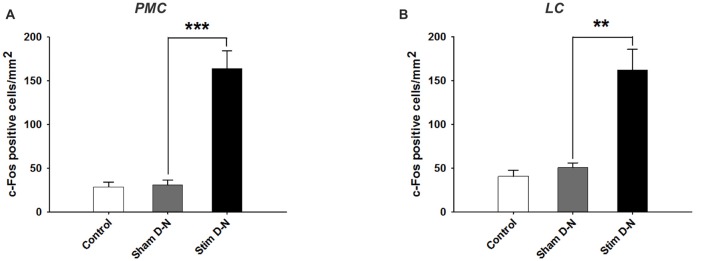
Expression of cFos immunoreactive cells in the PMC and LC. Cumulative data showing the means and SEM of cFos expression for D-N animals in **(A)** PMC and **(B)** LC. Control *n* = 6, Sham D-N *n* = 6 and Stim D-N *n* = 7. *t*-test, **p < 0.005 and ***p < 0.001.

**Figure 7:**
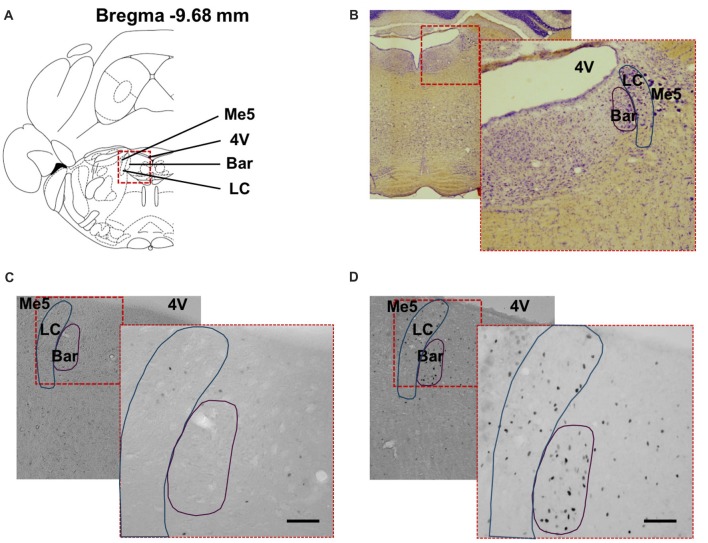
Distribution of cFos immunoreactive cells in the pontine structures. **(A)** Coronal diagram of the rat brain at corresponding Bregma level AP −9.68 mm (Rat brain atlas, Paxinos and Watson, 2007). Representative low power photomicrographs of coronal brain sections stained **(B)** with Nissl staining and for cFos showing the dorsal pons of **(C)** non-stimulated (Sham) rat and **(D)** stimulated rat (Stim) with bipolar stimulation electrode implanted in the bladder wall at the dome and neck levels. The higher power photomicrographs in the lower right corners show the PMC and locus coeruleus. 4V, fourth ventricle; PMC, Pontine micturition center or Barrington’s nucleus; LC, locus coeruleus; Me5, mesencephalic trigeminal tract. Scale bar = 150 μm.

The changes in cFos expression in the PMC and the LC were examined following electrical stimulation of the urinary bladder for the D-N group only, because this configuration showed the biggest effect size for the PAG. The numbers of cFos positive cells for both pontine structures was similar in control and sham animals (Control vs. Sham, *t*-test, NS, *p* > 0.05; Figures 6A,B). By contrast, electrical stimulation of the urinary bladder significantly increased the number of activated neurons in the PMC and LC (Sham vs. Stim, PMC and LC, ***p < 0.001, **p = 0.004, respectively; Figures 6, 7). Interestingly, whereas the activated neurons were distributed homogeneously throughout PMC, the cFos labeled neurons were mainly located in the dorsal part of the LC (Figure 7C).

### Effect of Electrical Stimulation of the Urinary Bladder on cFos Immunoreactivity in the Spinal Cord

**Figure 8:**
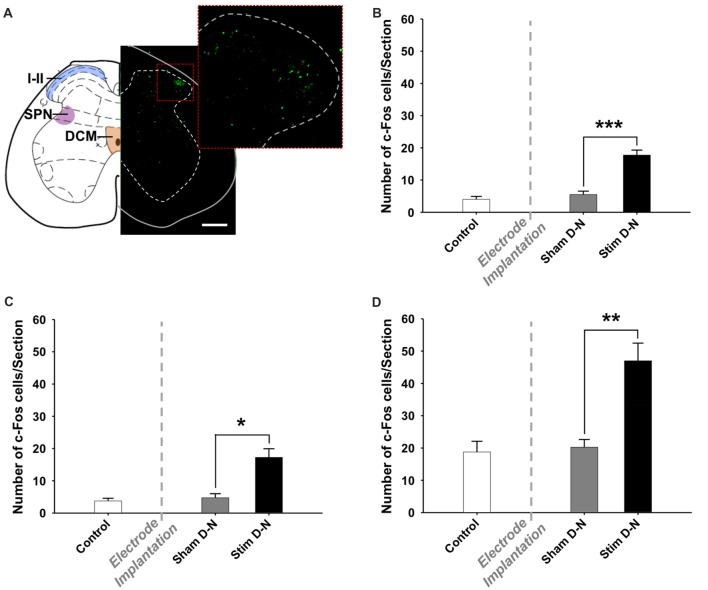
Expression of cFos immunoreactive cells in the lumbosacral spinal cord. **(A)** Coronal diagram of the rat lumbosacral spinal cord (L6) showing the location of the dorsal horn (i.e., laminae I and II), the sacral parasympathetic nucleus and the dorsal commissure (Rat brain atlas, Paxinos and Watson, 2007) and a corresponding photomicrograph showing a spinal cord section stained for cFos of stimulated rat with bipolar stimulation electrode implanted in the bladder wall in the D-N configuration. The higher power photomicrograph in the right corner shows the dorsal horn of the spinal cord. Abbreviations: I–II: laminae I and II, DCM: dorsal commissure, SPN: sacral parasympathetic nucleus. Scale bar = 200 μm. Cumulative data showing the means and SEM for the optical density of cFos expression for D-N group in the **(B)** SPN, **(C)** DCM and **(D)** I–II of L6-S2 spinal cord. Control *n* = 4, Sham D-N *n* = 4 and Stim D-N *n* = 4. *t*-test, *p < 0.05, **p < 0.005 and ***p < 0.001.

A quantitative assessment of cFos expression in the sacral parasympathetic nucleus (SPN), in the central canal region (DCM) and in laminae I and II of the dorsal horn (I–II) of the lumbosacral spinal cord (L6-S2) of D-N animals was done in a smaller sample size pilot study (Figure 8A; *n* = 2 per experimental group). No increase was seen in these structures between control and sham groups (Control vs. Sham, *t*-test, NS, *p* > 0.05; Figures 8B–D). However, cFos positive cells counts were significantly higher in stimulated compared to non-stimulated D-N animals (Sham vs. Stim, SPN, DCM and laminae I–II, ***p < 0.001, *p = 0.006 and **p = 0.004, respectively; Figures 8B–D).

### Macroscopic Evaluation of Urinary Bladder Tissue and Electrode Implantation

No sign of erosion, presence of excessive scar tissue or obvious change in tissue architecture in the urinary bladder and the surrounding tissues due to electrode implantation and chronic electrical stimulation performed during behavioral tests was revealed during *post-mortem* histological examination. Moreover, for all animals, the electrode wires remained correctly implanted in the bladder wall until the end of the experiments.

## Discussion

To our knowledge this study is the first that investigates the response of sensory afferent mechanosensitive fibers in the bladder in isolation in order to evaluate their distinct supraspinal neuronal response.

### Stimulation Parameter Choice

It has been shown by tracing studies that the PAG receives projections from the sacral spinal cord. Also, electrophysiologically this connection has been established in several studies. Yet, the studies performed mostly made use of nociceptive stimuli to prove this connection. The majority of afferent signaling from the bladder is however related to its filling status, which is signaled by mechanosensitization from stretch-receptors. We have spent a fair amount of work into optimizing the stimulus used to mimic mechanosensitization, because it is essential for the interpretation of the results and draw solid scientific conclusions (Buyle et al., 1998).

For the optimization of the stimulation parameters a duration of 30 s was used. Yet, for the final experiment we stimulated bladders for 1 h. We did not evaluate the effect of long-lasting stimulation on bladder contractility. Yet, it is known from electrical skeletal muscle stimulation, that frequencies in the range of 20–50 Hz produce smooth contractions, that could be sustained over long periods (Bhadra and Peckham, 1997; Doucet et al., 2012). Moreover, it has been shown, that high stimulation frequencies can cause the activation of motor neurons in the central nervous system. We aimed at triggering sensory fibers, which in turn could lead to the activation of motor neurons in the PMC. This will be discussed in more detail in the following paragraph. Low frequencies in combination with a short pulse width (around 500 μs) are less likely resulting in fatigue. In the case of 20 Hz stimulation it is unlikely, that a tetanus occurred, and that the bladder remained contracted during the stimulation phase. Tetany is generally known to occur at frequencies above 60 Hz. So, in the present experiment we expect, that continuous contraction and relaxation occurred during the stimulation period in the form of detrusor “twitches”.

Interestingly, somatosensory evoked potentials have been investigated in relation to different stimulation paradigms for anal sensory signaling. It has been shown, that low stimulation frequencies needed to be applied for longer durations in order to evoke the same somatosensory stimulation (Evers et al., 2014) than with higher frequencies. We therefore safely assume, that a stimulation period lasting 1 h activated central perceptions. Moreover, low to intermediate frequencies are considered more appropriate to stimulate Aδ-fibers of the LUT (Jiang, 1998; Langille et al., 2008).

Both charge transfer and heat dissipation due to electrical current can contribute to cell injury at the electrode-tissue interface (Crago et al., 1974). Thus, for minimal tissue damage during chronic stimulation, the amplitude should be set at a value causing minimum heat dissipation and charge transfer. Moreover, stimulation waveforms should be charge-balanced to reduce the possibility of tissue damage. In our case biphasic stimulation was considered most adequate. Intravesical pressure measurements showed that electrical stimulation with frequency of 20 Hz and amplitude of 2 mA was suitable and high enough to generate contractions of the urinary bladder, but these amplitudes did not cause voiding contractions.

As a critical note, one needs to realize that the simulation of a sensory stimulus is very challenging in rodents, because perceptions are nearly impossible to measure. However, studies investigating this in humans (i.e., functional magnetic resonance imaging) are very difficult to perform or miss the temporal or spatial resolution and therefore do not allow columnar analyses.

### Electrode Position

To evaluate PAG activity linked to urinary bladder sensation, the most appropriate parameter to stimulate the afferent nervous system of the LUT would be to initiate the sensation of bladder fullness by artificial bladder filling. However, functionally mapping of cFos needs to be applied carefully and critically (Dragunow and Faull, 1989), because cFos expression requires a prolonged and strong stimulation session, it is affected by anesthetic agents and can be induced non-specifically. Therefore, the introduction of a catheter for bladder filling, the measurement of bladder pressure and residual urine volume could induce cFos expression. This led us to opt for electrical stimulation of the urinary bladder in awake rats as a paradigm to study bladder sensation processing. Electrical stimulation is not a physiological stimulus, yet mimicking bladder sensation in a preclinical setting by electrical stimulation is a valid method. There is an added values of this stimulus for this type of sensory testing especially since it has been shown recently that afferent fibers detect bladder pressure or bladder wall stretch rather than bladder volume (Andersson, 2015; Janssen et al., 2017).

For the current set of experiments a custom-made bipolar electrode construction has been designed to be chronically implanted into the bladder wall of rats. The position for the implantation of the two wires within the bladder wall was determined in the line with the general distribution of mechanosensitive afferent fibers in the rat bladder. The myelinated Aδ fibers, described as afferent fibers responding to bladder filling and detrusor stretch and communicating filling sensation to the central nervous system, are mainly present in the smooth muscle layer of the urinary bladder (Iggo, 1955; Xu and Gebhart, 2008; Birder et al., 2010). Moreover, previous studies in rodents have revealed that the afferent fibers are notably more dense in the bladder neck and the dome (Grol et al., 2008). Accordingly, we placed the two wires at the level of the neck on both sides for the N-N group and at the level of the neck and the dome for the D-N group, between the outer muscular and serosal layers of the urinary bladder wall.

From a theoretical point of view in the N-N configuration the electrodes are closer to the location that has a high density of afferent neurons. It is unclear however whether sensory afferent neurons located at the bladder neck are Aδ or c-fibers. However, when stimulating a bigger area, which is the case in the D-N position, it is more likely that the whole detrusor muscle contracts rather than local contractions which leads to stretch-induced afferent activation of sensory neurons. However, as mentioned previously the stimulation parameters used—especially a pulse width of 0.5 ms—preferentially activate sensory afferents independently of their effect on contractility and or stretch. The difference in the density and eventually type of neurons in the different stimulation locations, might contribute to the differential activation of the rostral dlPAG and most columns of the caudal PAG, which did not respond to N-N but D-N stimulation. From a hypothetical point of view, activation stretch caused by bladder filling is exerting its effect on the whole detrusor muscle and does not occur at distinct locations such as the bladder neck. So theoretically speaking the D-N configuration seems to be more physiological. Based on these data not the density of neurons but rather the type and location in the bladder determine the activation of central nervous system structures. In the future, the identification of neurons that are responsible for PAG activation in transgenic animal models or in models with (transient) local lesion might reveal novel targeted treatment approaches for bladder disorders, with aberrant sensory processing.

### Behavioral Findings

There is a vast body of evidence that the PAG plays an important role in pain control (Young and Chambi, 1987; Bandler and Shipley, 1994; Rosen et al., 1994). Moreover, the dorsomedial and dorsolateral columns of the PAG have been demonstrated to be involved in anxiety-related behavior (dlPAG for the flight reaction and dmPAG for defensive behavior; Carrive, 1993; Keay and Bandler, 2001).

This raises the question whether electrical stimulation increases anxiety or pain and thus induced cFos expression in dorsal columns of the PAG. To exclude the possibility that cFos expression was induced by non-stimulation related effects, behavioral signs of pain and anxiety were analyzed in rats. There were no behavioral signs of pain nor did animals show anxiety-related behavior during the stimulation, which lead us to the conclusion, that cFos expression in the (dorsal) PAG, was not attributable to increased pain and anxiety.

However, in our study, an increase of urination frequency was found. In accordance with recent findings concerning afferents dysfunction in the development of bladder activity disorders such as OABS, the increased urinary frequency is likely to result from (hyper)excitation of the sensory system.

If—like applied in this experiment sensory afferent stimulation—results in a higher voiding frequency, this supports the hypothesis that OABS is in part related to faulty sensory signaling of bladder filling status. OAB is characterized by an increased frequency of voiding and urgency. Both are potentially related to faulty sensory signaling. From a neuromodulatory perspective it would be therefore interesting to test if it is possible by applying different stimulation parameters (i.e., high vs. low frequency) to ameliorate OAB symptoms by inhibiting sensory processing at the level of the bladder or even in the central nervous system.

Finally, before analyzing the results from cFos immunostaining, great care was taken to exclude that the observed changes were due to a displacement in the level of the electrode that would lead to unintentional stimulation of somatosensory receptors in the abdominal musculature and parietal peritoneum.

### Neuronal Activation in the Central Nervous System Upon Electrical Stimulation

Previous studies showed a key role for the PAG in the regulatory system of urinary bladder (Blok et al., 1997, 1998; Matsuura et al., 2000; Athwal et al., 2001; Taniguchi et al., 2002; Griffiths et al., 2005; Tai et al., 2009; Griffiths and Fowler, 2013). This structure is therefore seen as an interesting target to invoke treatment strategies to restore the normal function of the urinary bladder in urological disorders such as OABS. However, little is known about the relationship between the urinary bladder sensation and more specific PAG columnar activity.

**Figure 9:**
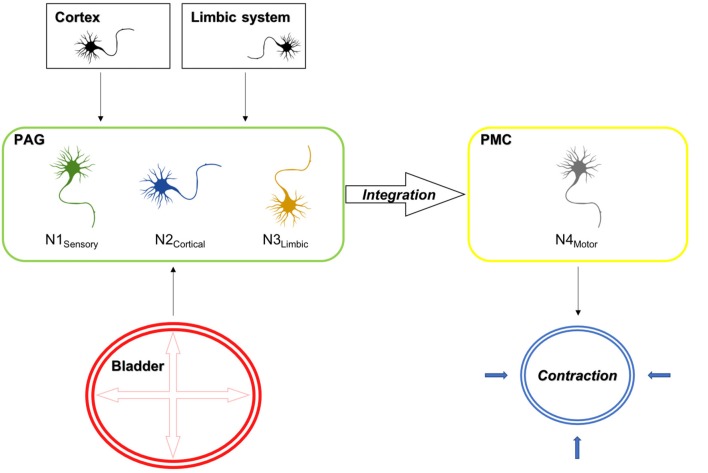
Electrical stimulation mimicking stretch of the detrusor muscle leads to the activation of sensory bladder afferents (Aδ fibers), which in turn activate sensory neurons in the PAG (N1 sensory). Besides, the PAG receives cortical and limbic input, which evaluates external circumstances, which either inhibit or facilitate voiding and activate cortical (N2 cortical) and limbic (N3 limbic) projection neurons in the PAG. The integration of these signals takes places in neurons projecting to the PMC. In turn the PAG releases its disinhibitory action on the PMC, which results in the activation of efferent neurons (N4 motor), which result in the corresponding action at the spinal cord, leading to detrusor contractions and the relaxation of the external urethral sphincter, which are prerequisites for voiding. Also shown is the activation pattern as assessed by cFos expression in the D-N configuration for the rostral and caudal PAG. Red areas indicate activation and green areas are not activated upon sensory afferent stimulation.

The findings of the current set of experiments with regard to supraspinal activation upon sensory afferent stimulation are summarized in Figure 9.

### Neuronal Activation of the PAG by Electrical Stimulation

Tracing studies have been performed earlier showing, that indeed there is a connection between the bladder and the PAG—more specially the ventrolateral column (Morrison, 1997). These results are in line with the current study. Whereas tracing studies are excellent to show neuroanatomical connection they cannot be used for functional connectivity assessment. The present study has the potential to combine neuroanatomical evidence with functional activation of neurons. To our knowledge there is no study investigating, which physiological stimulus activates neurons in brain structures such as the PAG. The studies that have been investigating (mainly by means of neurophysiological measurements) the activation of the PAG made use of noxious stimuli, which cannot be considered physiological. Considering that the PAG also plays a strong role in anxiety and pain-related functioning the use of noxious stimuli is biasing the results of earlier performed studies.

In control, Sham D-N and Sham N-N animals, few cFos immunoreactive cells were scattered throughout the PAG. These observations correspond to cFos basal expression and confirm that the presence of wires implanted in the bladder wall did not induce non-stimulation related cFos expression.

After electrical stimulation, the number of cFos positive cells was significantly greater than in non-stimulated animals. In the stimulated D-N group, cFos in the dorsomedial, lateral and ventrolateral regions of the caudal PAG was significantly increased compared to non-stimulated sham D-N group, with the biggest effect size in the vlPAG. Also, Marson (1997) showed, that the bladder projects to a very localized area in the vmPAG. Even though, the terminology used (ventromedial vs. ventrolateral) is different the location and pattern of labeled neurons they show is identical with the activated area that we found. In their study they used a tracer that was injected into the EUS. This distinct localization in combination with the knowledge that neurons are connected to the EUS offers opportunities for selective treatment options. Even though, we did not observe obvious clusters of neurons in other columns it might be interesting in future experiments to identify if there are neuronal subpopulations within the columns of the PAG.

A previous study on cFos expression following activation of bladder afferents by noxious stimuli (acetic acid) to the urinary bladder reported cFos positive cells in the lateral area of the vlPAG (Mitsui et al., 2003). The difference in columnar activation could be explained by the stimulation of different types of afferent fibers. Indeed, in the case of acetic acid infusion, mainly unmyelinated c-fibers respond to noxious stimuli (Aizawa et al., 2018). Electrically evoked (sub-micturition threshold) contractions generated increased activity through central pathways that control the urinary bladder, initiated by activation of sensory axons. In previous studies, various stimulation protocols of the LUT caused changes in the central pathways such as PAG, PMC and spinal cord (Birder and de Groat, 1992; Birder et al., 1999; Mitsui et al., 2001, 2003; Yamada et al., 2012).

It has been hypothesized, that the PAG receives information about the bladder filling status (Griffiths et al., 1990). To our knowledge this study is the first study proofing the concept that afferent bladder signaling is indeed received by the PAG and functionally activates neurons.

### Neuronal Activation of the PMC by Electrical Stimulation

The PMC has a well-documented role in the control of micturition and urinary bladder function (Noto et al., 1989, 1991; Fowler et al., 2008; Griffiths and Fowler, 2013). It is known that activation of PMC initiates a complete synergic micturition response by inducing detrusor contractions and the relaxation of the external urethral sphincter resulting in micturition. Since the ventrolateral and lateral caudal PAG are known to project specifically to the PMC and activate its premotor interneurons (Blok and Holstege, 1994; Fowler et al., 2008), activation of the PAG should result in dense cFos immunoreactive cells in PMC. Considering, that the PAG was activated minimally in the N-N configuration and the fact that activation of the PMC depends on PAG activation we investigated the expression of cFos in the D-N configuration only. CFos expression in the sham stimulated group showed a sparse and random distribution of cells expressing cFos whereas in D-N Stim group cFos positive cells were upregulated and consistently distributed.

Interestingly, we noticed in the nearby region, the LC, an increase in cFos immunoreactivity after electrical stimulation compared to non-stimulated animals presenting a low cFos level expression. Moreover, cFos positive neurons were primarily present in the dorsal half of the LC. We can conclude that the LC is activated upon bladder stimulation. Activation of LC neurons has been implicated in arousal and attention (Foote et al., 1983; Aston-Jones, 1985). In monkeys, cats and rats, sensory stimuli of various modalities can activate the LC-noradrenergic system (Foote et al., 1980; Aston-Jones and Bloom, 1981; Abercrombie and Jacobs, 1987). Visceral stimuli increase LC discharge rate and are important in initiating a forebrain response (Elam et al., 1986; Page et al., 1992). The LC has been previously identified to be connected anatomically to the bladder by means of tracing (Marson, 1997).

### Spinal Cord Activation

Previous studies have revealed that the caudal lateral, ventrolateral and dorsal divisions of the PAG receive many projections from the lumbosacral cord and that stimulation of the afferent bladder nerves in the rat evokes short latency potentials in the most caudal part of the PAG (Noto et al., 1991; Ding et al., 1997). Because neurons located in the laminae I and II, SPN and DCM of lumbosacral spinal cord project to the PAG, the visceroceptive inputs are likely to be propagated to the PAG through these spinal-PAG projections. We measured the cFos expression at the spinal levels of the L6-S2, since it corresponds to spinal entry sites of the pelvic nerve and to the regions of bladder afferent connections (Morgan et al., 1981; Ding et al., 1997; Xu and Gebhart, 2008). The present study demonstrated that bladder stimulation increases cFos expression in the superficial layers of the dorsal horn (laminae I and II), in the SPN and the central canal region in accordance with previous observations with noxious stimuli (Birder and de Groat, 1992; Birder et al., 1999; Mitsui et al., 2001). It should be noted that an increased cFos expression in the SPN may also be related to changes in the motor control of the vesical sphincters. The current study indicates strong neural activation consistent with mechanoceptive input in the spinal cord supports and validates the model of bladder electrical stimulation.

We optimized the parameters for the stimulation so that an intravesical pressure would occur caused by detrusor contractility in the absence of voiding. We applied the stimulation directly to the bladder muscle through the implanted electrode. There is no reason to believe that direct stimulation at the detrusor muscle itself causes the activation of efferent motor neurons in the central nervous system directly. Likely, efferent motor neurons were activated as a consequence of the bladder-spinal cord reflex network. One example of this is the guarding reflex, which occurs once there is a sudden rise in abdominal pressure in order to prevent leakage of urine resulting in the contraction of the EUS to prevent urine from leaking. Augmentations of intravesical pressure can lead to either the activation of the guarding reflex (short reflex loop at the level of the spinal cord) in order to prevent leakage of urine or the initiation of voiding (long reflex loop mediated via the brain).

In the spinal cord, the efferent system is exclusively located in the ventral horn, whereas the sensory part is located in the dorsal horn. Furthermore, motor neurons can be easily distinguished in the spinal cord due to their size and shape. It is indeed true that some motoneurons stained positive for cFos (see Figure 8A, which shows incidentally a motoneuron stained in the ventral horn). Due to the fact that we quantified cFos positive neurons in the dorsal part of the spinal cord only, we can surely say that these are related to sensory activity.

This means that we could: (i) proof that sensory activation took place in our setup; and (ii) exclusively focus on the sensory component by delineating and quantifying a neuroanatomical region, which is supposedly only involved in sensory information processing. As we were unable to assess if animals were aware of a sensory perception we proof a sensory component in our experiments by the activation of sensory neurons in a region of the spinal cord that is exclusively mediating sensory signals.

### Technical Considerations

For the purpose of identifying sensory processing upon sensory bladder related signals in the central nervous system, we used immunohistochemical detection of the cFos protein in the CNS of rats after urinary bladder electrical stimulation.

It is important to realize that cFos expression is a marker of neuronal activity but not a marker for the efferent result (such as the contraction of a muscle) or the effect on a distant neuronal target (such as the PMC, which is under the control of the PAG). The activation of inhibitory neurons (i.e., GABAergic interneurons) results in distinctive actions at the distant side than the activation of excitatory neurons. In this study, we did not differentiate between the type of neuron that was activated. However, we did follow-up on this question in a study, which was recently published studying the co-expression of cFos and neuronal markers in the PAG (Zare et al., 2018). Also, like described in the above diagram, due to input of various regions to the PAG, we cannot make a difference which neuronal activation resulted from afferent sensory or cortico-limbic inputs.

Staining of cFos does not allow to differentiate in temporal terms. Therefore, our study does not allow to differentiate the temporal sequence in which neuronal activation occurred. Even stronger rather than considering PAG columns as isolated entities it is possible that those interact and modulate their function. For instance, Noto et al. (1991) showed that stimulation of the pelvic nerve afferents induced evoked-potentials in the dorsal PAG. The optimal site for inducing bladder contractions was in the ventral PAG. This suggests, that afferent and efferent pathways are distinct in the PAG. Based on their results they suggest, that it is unlikely that the PAG is a relay station, but raise the possibility that the dorsal part of the PAG may have modulatory effects on micturition (Noto et al., 1991).

Moreover, it appeared that the location of the stimulation activated the caudal, but not the rostral (dl)PAG. The meaning of this differential activation is unknown. The focus with regard to functional differences in the PAG has mostly been on the columnar structure rather than functional difference in the rostro-caudal direction. It has been shown, that specific projections from one PAG column to other brain areas (i.e., rostral ventromedial medulla) differ in the rostro-caudal axis (Loyd and Murphy, 2009).

These regional differences cannot be investigated by exposure to noxious compounds because they are not applied in distinct parts of the bladder. Therefore, our experimental setup provides unique insights into the columnar activation of the PAG.

### Future Perspectives

To characterize and elucidate the function of the receptors involved in the activated neurons playing a role in the propagation of signals generated by the bladder, additional investigations by immunohistochemistry and *in vivo* electrophysiological experiments are required. Since several levels of the central nervous system are involved in the control of micturition, a cFos study in structures such as the prefrontal cortex, the insula and the anterior cingulate cortex should be considered to evaluate their potential influence in the processing of urinary bladder sensation elicited by electrical stimulation.

A deep understanding of complex neural circuits controlling the urinary bladder can be offer opportunities for novel modalities for OABS. Among the innovative therapeutic approaches to restore the correct functioning of the urinary bladder in OABS patients, deep brain stimulation (DBS) seems to be an attractive alternative treatment modality (Green et al., 2012; Stone et al., 2015).

## Author Contributions

CM, AJ, LB, YT and GK: designing research studies. CM: conducting experiments. MC, RH and SS: acquiring data. CM, RH, SS and AZ: analyzing data. YT and GK: providing reagents. CM, SS and GK: writing the manuscript. CM, RH, AZ, AJ, LB, SS, YT and GK: reviewing the manuscript.

## Conflict of Interest Statement

CM is a Marie Skłodowska-Curie postdoctoral fellow. The other authors declare that the research was conducted in the absence of any commercial or financial relationships that could be construed as a potential conflict of interest.

## Supplementary Material

FIGURE S1 Schematic diagram of the dorsomedial (dm), dorsolateral (dl), lateral (l) and ventrolateral (vl) longitudinal neuronal columns of periaqueductal gray matter (PAG) along the rostro-caudal axis (from left to right on the figure) and associated representative photomicrographs of coronal brain sections stained for cFos and delineated for the PAG. Note that the dark spot on bottom left side of photomicrographs is due to an imperfection in the 4× objective but did not interfer with delineation of the columns and the quantification of the cell number performed on photomicrographs taken with 10× magnification.

FIGURE S2 Methodological process for the quantification of cFos immunoreactive cells. Acquisition of the photomicrographs from the region of interest with a low magnification (4x) **(A)** for the delineation of the PAG **(B)**. **(C)** Delineation of the columns of the PAG with a higher magnification (10x). **(D)** Application of a thresholding method on the delineated sections. **(E)** Manual counting of cFos positive cells.
