# Supplementary figures and images for: Neuronal Activation in the Periaqueductal Gray Matter Upon Electrical Stimulation of the Bladder
Source: Front Cell Neurosci. 2018 May 18;12:133. doi: 10.3389/fncel.2018.00133 (PMC5968116; doi:10.3389/fncel.2018.00133)

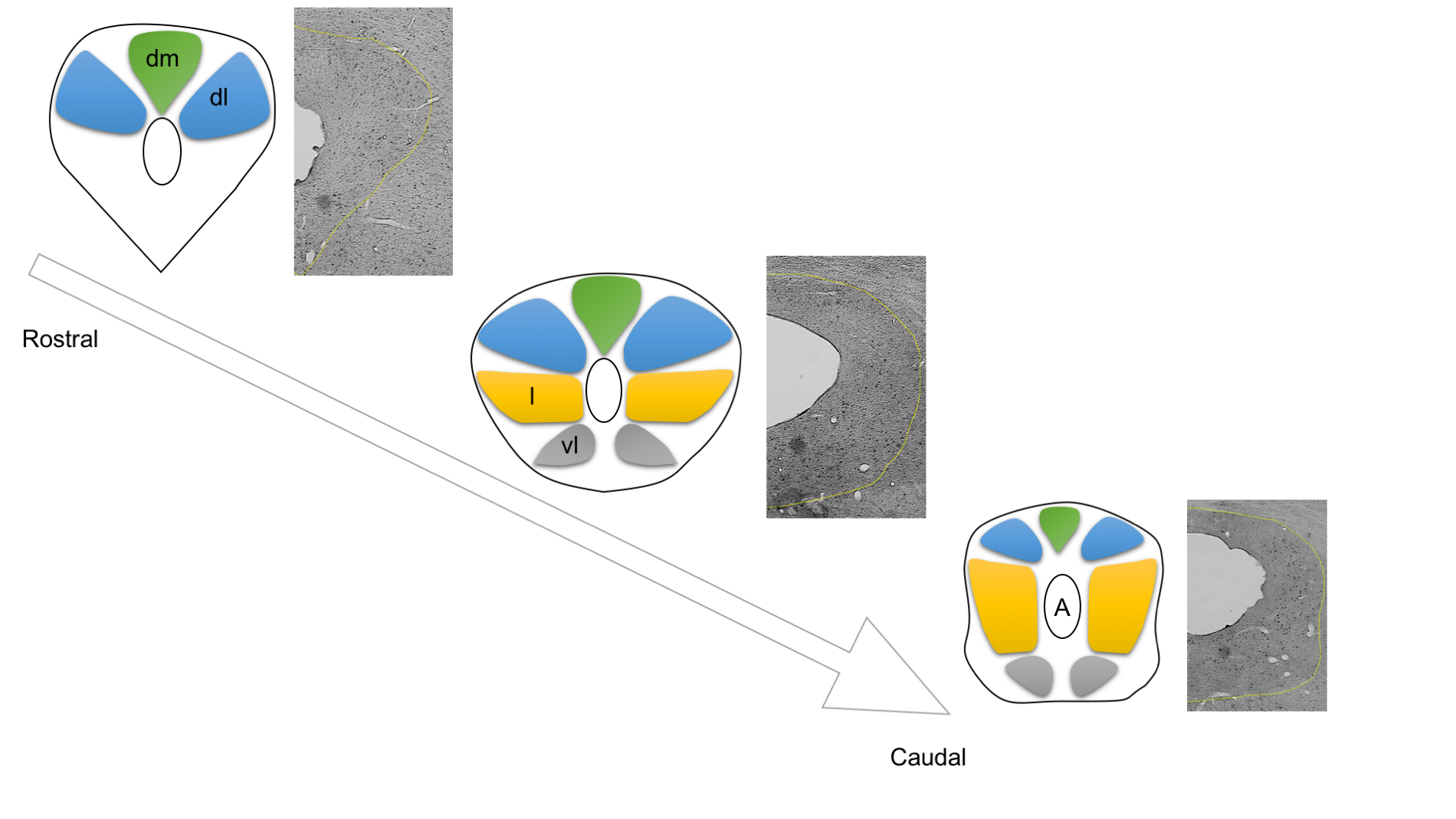

Supplement: FIGURE S1 — Schematic diagram of the dorsomedial (dm), dorsolateral (dl), lateral (l) and ventrolateral (vl) longitudinal neuronal columns of periaqueductal gray matter (PAG) along the rostro-caudal axis (from left to right on the figure) and associated representative photomicrographs of coronal brain sections stained for cFos and delineated for the PAG. Note that the dark spot on bottom left side of photomicrographs is due to an imperfection in the 4× objective but did not interfer with delineation of the columns and the quantification of the cell number performed on photomicrographs taken with 10× magnification. [file Image_1.TIFF]

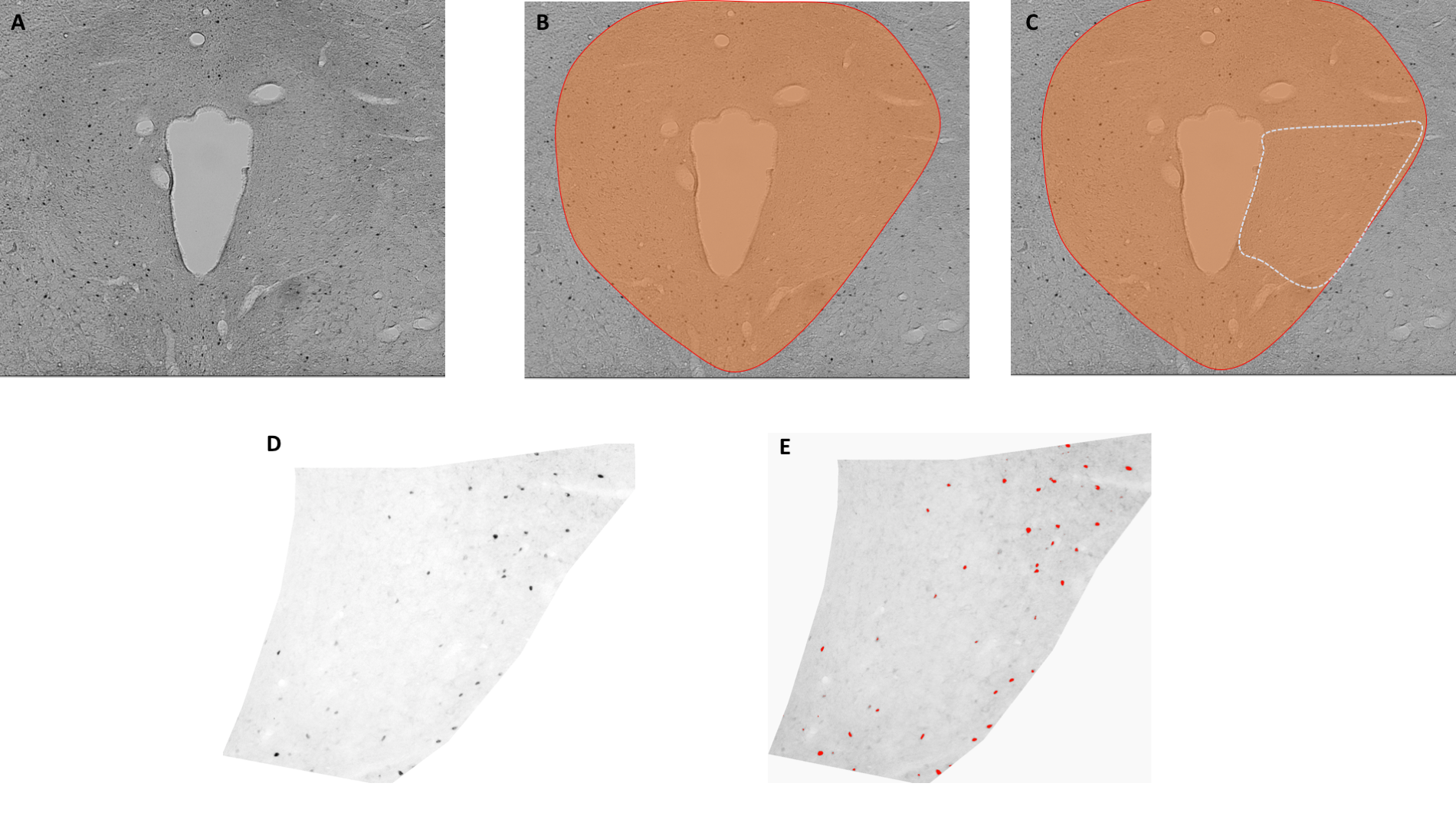

Supplement: FIGURE S2 — Methodological process for the quantification of cFos immunoreactive cells. Acquisition of the photomicrographs from the region of interest with a low magnification (4x) (A) for the delineation of the PAG (B). (C) Delineation of the columns of the PAG with a higher magnification (10x). (D) Application of a thresholding method on the delineated sections. (E) Manual counting of cFos positive cells. [file Image_2.TIFF]
